# Digital Health Literacy and Information-Seeking Behavior among University College Students during the COVID-19 Pandemic: A Cross-Sectional Study from Denmark

**DOI:** 10.3390/ijerph19063676

**Published:** 2022-03-19

**Authors:** Carsten K. Bak, Jeanne Ø. Krammer, Kevin Dadaczynski, Okan Orkan, Jesper von Seelen, Christina Prinds, Lene M. Søbjerg, Heidi Klakk

**Affiliations:** 1 Department of Research of Applied Science, University College South Lembckesvej 7, 6100 Haderslev, Denmark; jokr@ucsyd.dk (J.Ø.K.); jvse@ucsyd.dk (J.v.S.); cpri@ucsyd.dk (C.P.); lmso@ucsyd.dk (L.M.S.); hkeg@ucsyd.dk (H.K.); 2 Department of Health Science, University College South Denmark, Degnevej 16, 6705 Esbjerg Ø, Denmark; 3 Department of Nursing and Health Science, Fulda University of Applied Science, 36037 Fulda, Germany; kevin.dadaczynski@pg.hs-fulda.de; 4 Center for Applied Health Science, Leuphana University of Lueneburg, 21335 Lueneburg, Germany; 5 Department of Sport and Health Sciences, Technical University Munich, D80992 Munich, Germany; orkan.okan@uni-bielefeld.de; 6 Department of Clinical Institute, Faculty of Health Sciences, University of Southern Denmark, Kløvervænget 10, DK-5000 Odense C, Denmark; 7 Research Unit of Exercise Epidemiology, Institute of Biomechanics and Sports Science, Faculty of Health Sciences, University Southern Denmark, Campusvej 55, 5230 Odense M, Denmark

**Keywords:** digital health literacy, COVID-19, Denmark, information seeking, university college students, social network

## Abstract

The COVID-19 pandemic and the concomitant infodemic have emphasized the importance of digital health literacy (DHL) to global public health research and practice. The aim of this study was to examine information-seeking behavior, the ability to find, understand and deal with health information among university college students in Denmark and/in addition we wanted to examine the impact of their close social network on students’ ability to find and understand health information. This research was carried out as part of the COVID-HL university student survey by using a uniform questionnaire consisting of elaborated scales. Data were collected from a cross-sectional survey conducted at University College South during 4 weeks in April and May 2020. To capture DHL, four subscales of the DHL instrument were adapted to the pandemic context. A total of 59.9% of the students have sufficient DHL—most students find it rather easy to find information and are satisfied with the information they find on the internet. However, some (28.1%) students find it difficult to judge the quality and reliability of the information. Students with a sufficient level of DHL are more likely to seek information through search engines and websites of official institutions, while students with a limited level of DHL more often use social media for health information. Students with sufficient DHL more often share health information and less often ask for support in their network

## 1. Introduction

Severe acute respiratory syndrome coronavirus 2 (SARS-CoV-2) was first reported in a Chinese Province in December 2019 and has since evolved into a global pandemic with catastrophic humanitarian consequences as well as politically, economically, and socially [1]. The first individual with COVID-19 in Denmark was registered on 27 February 2020. During the pandemic, information, including dis- and misinformation, was spread about COVID-19 on the internet, via social media and other digital sources concerning topics such as diagnosis, protective behaviors, statistics, and recommendations from experts [2,3,4]. The crisis has highlighted the importance of the ability to acquire and apply health information and to change behavior to be able to avoid infection and spread of the virus [2,3,4]. At the same time, it is paramount that governments and health authorities implement a well-reasoned and evidence-informed health communication strategy to provide valid and reliable health information concerning the COVID-19 pandemic and support individuals to make health-promoting decisions [5,6]. In this context, health literacy must be understood as a social vaccine, a critical tool in the emergency toolbox of public health action to counteract the effects of the pandemic [5,7].

Along with the COVID-19 pandemic came the infodemic, which is the short version of “information epidemic”, which has proven to be a serious public health thread. The infodemic portrays the rapid spread of valid and invalid information on the internet or via other digital sources [5]. Infodemic appears now as a rather new term, but it was first introduced during the SARS epidemic in 2003 [6] and is now being used to illustrate the rapid production and consumption of information during the COVID-19 pandemic [8,9,10]. In other words, we are not only witnessing a ‘pandemic’ but also an ‘infodemic’ [11]. Sylvie Briand, director of Infectious Hazard Management at the WHO’s Health Emergencies Programme and architect of the WHO’s strategy to counter the infodemic, emphasized, “We know that every outbreak will be accompanied by a kind of tsunami of information, but also within this information you always have misinformation, rumors, etc. We know that even in the Middle Ages there was this phenomenon. But the difference now with social media is that this phenomenon is amplified, it goes faster and further, like the viruses that travel with people go faster and further” [11].

Health literacy concerns the ability to access, understand, appraise, and apply information to make informed decisions about health [12]. According to European research, more than one-third of the general population are at risk of low health literacy [13]. This is of special concern during a pandemic, as people with low health literacy have been found to show lower awareness, knowledge and protective behavior, which might result in a greater risk of COVID-19 infection [5,14,15,16,17,18]. Often, they do not understand important details and misinformation spreads very quickly. Decisions based on, e.g., dis- and misinformation can have major impacts on people’s health and safety [2]. A German study on coronavirus and COVID-19-related HL among adults (>16 years) found that 50.1% of the population have trouble in dealing with the coronavirus and COVID-19-related health information in their daily lives [14].

Abel and McQueen argue that a major challenge during COVID-19 is how the individual can integrate information into personal behavioral actions. Critical health literacy is needed, as it is not enough to just know about the risks (e.g., ‘functional’ health literacy). It is important for individuals to know how to critically assess overwhelming information [8], especially when it comes to the spread of fake news on the internet as fake news poses a severe threat to people’s health and safety [2].

Digital health literacy (DHL) has been defined as “the ability to seek, find, understand, and appraise health information from electronic sources, and apply the knowledge gained for preventing, addressing, or solving a health problem” [12]. However, students’ digital health literacy has not been well researched, especially not in relation to crises such as communicable disease epidemics and pandemics. While the past two years have demonstrated the consequences for personal social life and society (economically and politically), how university college students face the challenges of this infodemic and pandemic and how does this affect their information-seeking behavior have to be considered as well [19,20].

COVID-HL network publications (www.covid-hl.eu) have investigated different consequences of the COVID-19 pandemic via surveys. In an Australian study [21], most undergraduate students (65.5%) reported a low/very low level of psychological well-being and academic experience. Another study from Hong Kong and Macao [22] found that students with low SES are likely to require support to access critical resources/tools to improve their DHL.

Studies from European countries, e.g., Germany [20] and Slovenia [23], found that half of the students have trouble judging the reliability of digital information and judging the reliability of digital information. The German study found that 58.5% of students have limited HL [20]. This might also be due to a lower degree of digitalization in Germany and other European countries compared to a rather high degree of digitalization in Denmark. A study surveying US college students found that students with high DHL were more positive about being vaccinated against COVID-19, whereas those with lower DHL were more likely to consider the pandemic as an overreaction [14]. A Portuguese study of university students found significant gender differences related to difficulty with health information. Portuguese male students reported significantly low health literacy [19].

Our cross-sectional Danish study is a contribution to the ongoing international studies on university students’ DHL [19,20,21,22,23,24,25] and makes it possible to examine whether the findings from other countries can be replicated in our Danish sample. Based on the high degree of digitalization in Denmark, we hypothesize that Danish students have higher DHL compared to, e.g., the Germany sample with a lower degree of digitalization [20].

Health literacy research has mostly focused on individual skills and has thereby neglected the importance of help and support in finding and understanding health information [26]. Sentell and colleagues performed a pilot study [27] measuring health literacy in a social context among vulnerable new mothers in Hawaii and found that social networks were critical to health information dissemination and interpretation. Individuals with low HL and DHL need to find other people in their close social network to help them assess and understand health information from different sources. Longitudinal qualitative studies have investigated the impact of close social networks, e.g., family and friends, through ‘health literacy mediators’ and the concept of ‘distributed health literacy’ [26,28,29].

The aim of this study was to investigate how university college students in Denmark search, find and use digital health-related information related to the COVID-19 pandemic. As part of our investigation, we also include the importance of social networks in students’ health-seeking behavior.

The specific objectives for this article were to assess:

- DHL across social characteristics such as subjective social status, gender, age, and education;
- The preferred sources, platforms, and topics for seeking information on COVID-19;
- How students judge the validity and sufficiency of the information they encounter;
- Use of social media and DHL among students;
- The association of social networks and DHL among students.

## 2. Methods

### 2.1. Study Design and Target Group

**Figure 1:**
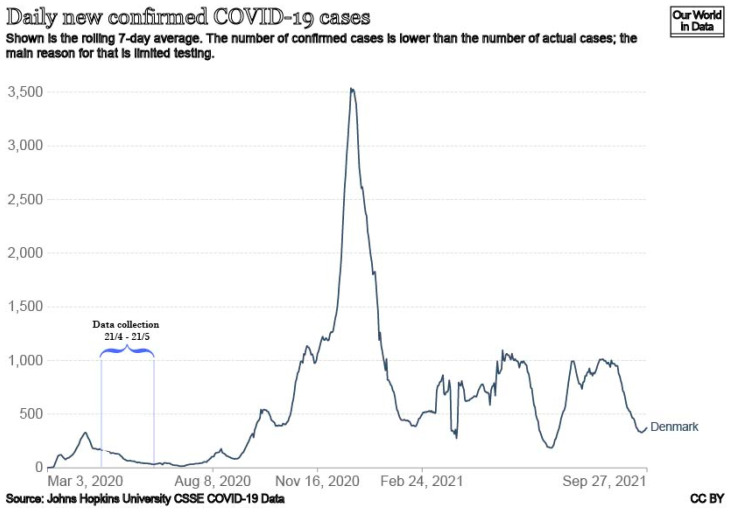
Daily confirmed COVID-19 cases.

A cross-sectional survey was conducted at University College South (UC SYD) during 4 weeks in April and May in 2020 (Figure 1). Figure 1 shows the daily confirmed COVID-19 cases in Denmark in spring 2020 and data collection was carried out in the period 21 April–21 May 2020. At the Prime Minister’s first press conference on 11 March 2020, concerning COVID-19 restrictions, all public employees performing non-critical functions and pupils and students from all educational institutions were sent home. Private institutions and firms were urged to do the same.

The UC SYD contributes as a partner in an international COVID-19 health literacy consortium with participants from more than 70 countries around the world (www.covid-hl.eu). The collaboration project “COVID-19 Health literacy (COVID-HL)” was initiated by researchers at the Public Health Centre at Hochschule Fulda (PHZF), the Interdisciplinary Centre for Health Literacy Research at Bielefeld University and the Health Literacy Chair at Technical University Munich in March 2020 with the aim of investigating students’ digital health literacy during and related to the COVID-19 pandemic.

We used a translated Danish version of the English questionnaire developed by the German coordination office of the COVID-HL network [24]. The questionnaire provided consists of already scientifically validated questionnaires and scales and was semantically adjusted for the special context of health information in relation to COVID-19 [24]. The international survey consists of 37 questions covering sociodemographic information, e.g., sex, age, region, and university college study direction (q 1–7); social position, living situation, and attitudes towards the future (q 1–7); information-seeking behavior/digital health literacy (q 13–23); social network and sharing information (q 25–26); health (q 27–28); well-being 10 (q 27–37). Scale documentation and validation of the German questionnaire are available here [14,15] and available as open access in German and English versions (https://pub.uni-bielefeld.de/record/2942920).

### 2.2. Participants—Recruitment and Distribution

The survey “Students digital health literacy during COVID-19 pandemic” is an online self-administered questionnaire conducted by researchers from the Research Department of the UC SYD. All university college students from the UC SYD (n = 5533) were invited to participate by student email and social media. After one and two weeks, a reminder was sent by email to increase the participation rate. The participants used a link to fill out the questionnaire online. The task was estimated to take approximately 15 to 20 min.

### 2.3. Measures

#### 2.3.1. Social Characteristics and Economic Variables

Participants were asked about their sex, age, education/current study program, semester, subjective social status [30]. 

Age (years) was divided into age groups; sex into male, female, and diverse; education/current study program (all bachelor’s degree with 15 different subjects) into four main groups (1) education (teachers), (2) health education (nurses, midwifes, medical laboratorian technologists/laboratory technicians, occupational therapists, physiotherapists, and dietitians), (3) society and administration (social workers, tax and public administration, and communication) and (4) other (further education and post bachelor’s degree).

Five students reported ‘diverse’ sex. Due to this small number, statistical analysis was not possible on these individuals as a solitary group.

Subjective social status: As in previous studies, respondents were categorized into three groups: low SSS (1–4), medium SSS (5–7), and high SSS (8–10) [20].

#### 2.3.2. Digital Health Literacy Questionnaire 

Digital health literacy was evaluated with the validated Digital Health Literacy Instrument (DHLI) [31]. The DHLI items were adapted to the current context by specifically addressing digital health literacy during the COVID-19 pandemic (i.e., When you search the internet for information on the corona virus or related topics, how easy or difficult is it for you to…). The DHLI contains seven subscales, each including three items to be answered on a 4-point Likert scale (i.e., 1, very difficult; 4, very easy). In the COVID-HL survey, five of these seven subscales were used and assessed self-reported skills in the areas of:

1. Searching and finding health information on the internet,
2. Creating and sharing of health information on the internet,
3. Assessment of the quality of health information on the internet,
4. Determining the everyday relevance of health information on the internet, and
5. Handling of personal information and data protection on the internet.

As proposed by the COVID-19 Network [15], we only used four of the seven subscales to calculate levels of DHL. These subscales were (1) information searching, (2) adding self-generated content, (3) evaluating reliability, and (4) determining relevance. All data on DHL and information-seeking behavior will be presented descriptively and analytically by level of DHL (limited vs. sufficient). Table 1 shows the questions used in the analysis of DHL. See also Table 1 for two questions and question options to assess ‘social network’ (q 25–26).

#### 2.3.3. Statistical Analyses

Initially, all data on digital health literacy and the use of internet sources were analyzed descriptively. Furthermore, we performed bivariate analyses by using the chi-squared test to determine whether there are any differences between participant sociodemographic characteristics and the two levels of digital health literacy (limited vs. sufficient). The DHL subscales and overall DHL were dichotomized based on a median split to allow bivariate analyses. The participants below the median were classified into the group with limited DHL, and the participants above or exactly at the median were classified into the group of sufficient DHL. The subscale ‘protecting privacy’ was not dichotomized or included in the calculation of overall DHL due to low internal consistency.

A t-test was used to determine possible statistical differences between sufficient and limited overall DHL and the DHL subscales in frequency of use of internet sources used for web-based health information. These analyses were supplemented by regression analyses to estimate relationships between DHL as a dependent variable and internet sources used as independent variables.

For all analyses, alpha was set at 0.5 and a *p*-value ≤ 0.05 was considered statistically significant. With large sample sizes, an association may be statistically significant but still have a negligible effect, hence why the strength of the association was determined by Cramér’s V (for the chi-squared test) or Cohen’s d (for t-test). For Cramér’s V, the effect is considered small at ≥0.1, medium at ≥0.3, and large at ≥0.5, according to Cohen [27]. For Cohen’s d, the effect is considered small at ≥0.2, medium at ≥0.5, and large at ≥0.8 [32]. When interpretating the results, a value below ‘small’ for either Cramér’s V or Cohen’s d means the difference is practically negligible, although statistically significant.

Associations between overall DHL and sources used for information seeking were analyzed using logistic regression. Potential confounders (gender, age, subjective social status, year of study, and course) were included in the multivariate logistic regression, and an adjusted odds ratio (OR) with a 95% confidence interval (CI) is presented.

For all the statistical analyses, SPSS (Statistical Package 27) was used.

#### 2.3.4. Ethical Considerations

Projects of this type do not require ethical approval by national or regional boards in Denmark. This project has been approved by the internal secretary for research projects in the UC SYD. Data are anonymized, stored securely in ‘Science Data’ (Danish national data storage) and hence not in conflict with GDPR requirements.

## 3. Results

Participant sociodemographic characteristics are shown in Table 2. A total of n = 5533 students from the UC SYD received a link to complete the self-administered questionnaire. In total, 1518 students (1266 female, 247 males and 5 diverse) participated and n = 1106 (73%) completed the full questionnaire, while n = 412 (27%) provided some answers. The four main categories of study direction were education, health, society and administration and others. Half of the respondents studied education (teachers) (n = 751, 49.5%), one-third were health education students (n = 470, 31%), the rest were society and administration students (n = 264, 17.5%), and less than two percentage responded “other” (n = 26, 1.7%). Most of the study participants (88.6%) receive state education support (SU), either as their sole income (28%) or supplemented with study loans (22%) and/or student jobs (33%). The majority are satisfied with their financial situation or very satisfied (94.2%) and 5.8% find their financial situation unsatisfactory. The majority live with others and 20.5% live alone. The mean age of the participants is 28.4 years (SD 8.4 years, range 18–60 years). A total of 87.6% of the participants were born in Denmark. Most university college students reported medium subjective social status (SSS).

### 3.1. Across Social Characteristics

Stratified by social characteristics, no statistical significance was found for overall DHL. Looking at the distribution of limited vs. sufficient groups for the DHL subscales, few significant differences were found for the subscale ‘adding self-generated content’, with Cramér’s V indicating a small effect. Younger respondents (<26 years) reported more difficulty in adding self-generated content (ꭕ^2^ = 16.03, *p* < 0.001, V = 0.12), while respondents with higher SSS reported less difficulty (ꭕ^2^ = 10.56, *p* < 0.005, V = 0.1).

#### Preferred Sources, Platforms and Topics for Information Seeking

**Figure 2:**
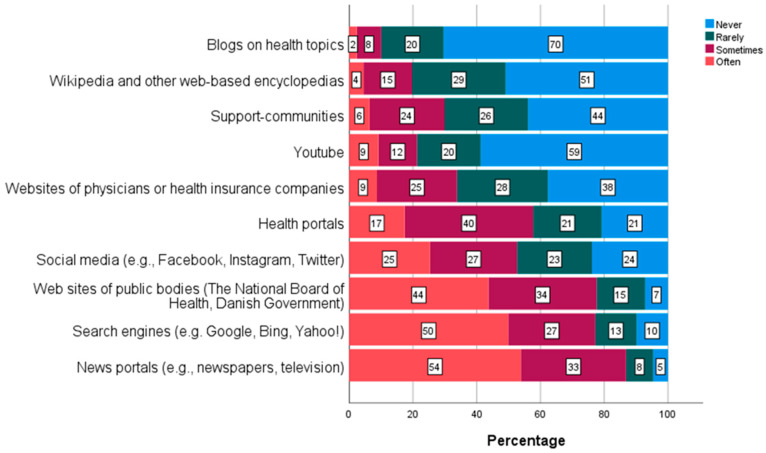
Frequency of use of internet sources for web-based health information seeking (n = 1468 to n = 1513), Pct.

As depicted in Figure 2, preferred platforms among students were (i) search engines (e.g., Google, Bing or Yahoo), (ii) websites of public bodies or (iii) news portals (e.g., newspapers or TV stations) by 77%, 78% and 87%, respectively, indicating using these sources “often” or “sometimes”. Additionally, social media (e.g., Facebook, Instagram and Twitter) were used often or sometimes by 52%. Wikipedia, YouTube, Guidebooks and websites for doctors or health insurance companies were used to a lesser extent (19%, 21%, 29% and 32%, respectively), whereas blogs on health topics were seldom used for information seeking (2% indicated often and 8% indicated sometimes).

The analyses concerning possible differences between the levels of DHL and the internet sources used for information search showed that respondents with sufficient overall DHL used social media significantly less, although the difference was found to be practically negligible (limited, mean 2.75; sufficient, mean 2.41, *p* < 0.001, d = −0.16). Regarding the DHL subscales, statistically significant results were found for ‘information search’, ‘evaluating reliability’, ‘adding self-generated content’, but when taking Cohen’s d into account, the analyses showed that respondents with sufficient ability to evaluate reliability used social media significantly less than respondents with limited ability (limited, 2.82; sufficient, (2.40), *p* < 0.001, d= −0.21). Furthermore, regression analyses (multivariate) showed that only the frequency of social media use could predict the risk of having limited overall DHL. Respondents who never/rarely used social media for information seeking had a lower risk of having limited overall DHL (adj. OR 0.537, *p* < 0.001).

**Figure 3:**
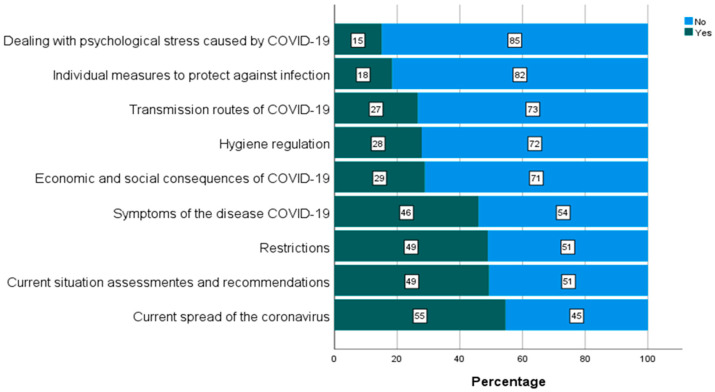
Internet search queries related to COVID-19 (n = 1518). Pct.

The most preferred topic to search for (out of ten given) was “current spread of the coronavirus” (55%) followed by topics on current situation and recommendations, restrictions (e.g., exit restrictions and stay-at-home orders) and symptoms of COVID-19 (49%, 49% and 46%, respectively). The results are shown in Figure 3.

### 3.2. Ability to Find Information

Most students find it easy/very easy to use the proper words (92.7%) to find information but one out of five students (18%) find it difficult/very difficult to find the exact information. A total of 15.2% find it difficult/very difficult to choose between all the information. Most university college students are generally satisfied with the information they find online about coronavirus (70.8%), and only 4.8% are dissatisfied/very dissatisfied. A total of 16.1% are partly satisfied and 8.3% are very satisfied. See Table 3.

Most students find it easy/very easy to express opinions, thoughts, or feelings in writing (76%) or write messages that are understood (74.4%). However, 28.1% find it difficult/very difficult to decide if information is reliable, verified and up to date and comes from official sources.

Most students believe that they can apply information about coronavirus in their daily life and that it is applicable for them. Many students find it difficult/very difficult to judge who can read the messages they post on social media and protect their privacy.

### 3.3. Support from Social Network and Sharing of Health Information

Table 4 includes analyses concerning whether students’ get help from their close social network (e.g., family and friends) and the two levels of DHL (limited vs. sufficient). Statistical significance was found for overall DHL, although Cramér’s V indicates the difference to be practically negligible. 

When looking at the distribution of limited vs. sufficient groups for overall DHL in relation to whether students received help from their close social network to find information about COVID-19, significant results were found. Among students who tend to get help from their close social network, a higher percentage of limited DHL was found (ꭕ^2^ = 9.81, *p* = 0.002, V = 0.11). Among those who often received help, a higher percentage was found compared to among those who sometimes or rarely received help (ꭕ^2^ = 11.95, *p* = 0.008, V = 0.12).

## 4. Discussion

The present study has examined how university college students in Denmark during the first cycle of COVID-19 pandemic search, find and use digital health-related information by assessing levels of DHL across sociodemographic factors and preferred information-seeking platforms. We also included an analysis of the importance of social networks in students’ information-seeking behavior.

Our cross-sectional Danish study is a contribution to the ongoing international studies on university students’ DHL [21,22,23,24,25] and the findings from this study provide new knowledge about the vulnerability of students in crisis situations, such as the COVID-19 pandemic, when high DHL is necessary. This knowledge can inform the designers of interventions aiming at strengthening DHL as a social vaccine [7] to avoid the further spread of COVID-19 among students.

In this study among university college students, over half of our sample have an overall *sufficient* level of DHL (59.9%). Findings from other studies show more students with a limited level of DHL, e.g., among US college students [14] and students from Germany [20], where 58.5% have limited HL, which points to many risky sources of digital health information and pitfalls of misinformation.

In the analysis concerning participant sociodemographic characteristics and the two levels of digital health literacy (limited vs. sufficient), no statistical significance was found for overall DHL. Only a few statistical results were found on the DHL subscales when stratified for social characteristics.

More than half of the students have sufficient DHL and most of the university college students in Denmark find it easy/very easy to find information and they are satisfied/very satisfied with the information they find on the internet. The preferred platforms are different news portals, search engines as, e.g., Google and Yahoo, and websites of public bodies where the students ‘often’ search for health information. However, one out of four students often use social media (e.g., Facebook and Twitter) for health information. It is very easy to access many different types of information, but it is a more difficult task to find information of high quality and reliability, navigating through information of various complexity, which presents an interesting paradox [23].

Almost one out of three students find it difficult/very difficult to decide if the information is reliable, verified and up to date and comes from official sources. This result is in line with recent similar studies. A Slovenian study [23] shows that one out of two students (49.3%) have trouble judging the reliability of digital information and one out of three students experience difficulty selecting among the information. This is seven percent more than found in a German COVID-HL survey [20]. Among Danish university college students, almost one student out of three (28.1%) reported experience difficulty determining the reliability of digital information. Many Danish students find it difficult (45.2%) to judge who can read the messages they post on social media and protect their privacy. This points to the importance of health literacy, as suggested by Abel and McQueen [8], highlighting that it is not enough to know about risks. It is equally important to be able to critically assess the large variety of information available from the internet and the validity of different sources. The study of US college students showed that students with high DHL were more willing to be vaccinated against COVID-19. Those with lower DHL were more likely to consider the pandemic an overreaction [14].

Students with lower DHL are more prone to using social media frequently and are thus at risk of being exposed to more misinformation than those with higher DHL who use social media less. Similar results were reported by other countries from the COVID-HL survey such as Portugal [19], Germany [20] and Slovenia [23]. Our analysis of the impact of help from close social network relations showed that students with sufficient DHL helped their close social network more in assessing the credibility of health information on the internet and from health authorities, and in giving advice on, e.g., maintaining a healthy lifestyle. Students with limited DHL more often get help from their close social network, which indicates the importance of including social network in health communication strategies and interventions. The COVID-HL findings from Hawaii show that, on average, respondents discussed health with 4–5 people, which did not vary by HL or DHL measures [14].

Difficulty in determining the reliability of information is connected to preferred information sources and therefore we suggest that DHL could be a part of a health communication strategy at university colleges and universities, as suggested by Patil and colleagues [14]. This could be implemented in teacher training and education efforts to support overall strategies to increase students’ DHL before they leave, e.g., university college. It should also be part of lectures where students are involved in critical thinking and learn how to separate accurate from false and misleading information [14].

This study has limitations. It is a cross-sectional study using self-reported data from Danish university college students conducted during the first wave (from 21 April to 21 May 2020) of the COVID-19 pandemic outbreak in Denmark. In this period, information about COVID-19 might have been easier to understand as it was presented and supported by the government and public health agencies. In the first period in spring 2020, the daily confirmed COVID-19 cases were not as high as in Autumn 2020 and this situation might have contributed to the overall high satisfaction with information about COVID-19.

Further empirical research on students’ DHL might clarify other dimensions of DHL for information-seeking behavior than the dimensions used in this study. Differences in social norms, health literacy in different population groups, e.g., children, patients, vulnerable people, people with chronic conditions and in rural settings, are relevant topics to investigate.

Furthermore, we suggest future HL research re-visit the premises for dichotomization of the DHL scale into limited vs. sufficient DHL by median split, as we found that more than 76% in our sample responded easy or very easy to the question concerning DHL. We suggest applying a more logical dichotomization of easy/very easy vs. difficult/very difficult, rather than the suggested mathematical theoretical distribution with median split as it seems very illogical that quite a large proportion of those who have answered easy/very easy in the subscales will be classified with limited DHL (see numbers and proportions for levels of DHL in Table 2 vs. Table 3).

Future research should incorporate more scales to widen the scope of the analysis, for instance the importance of their close social network (distributed health literacy) for understanding information and critical application of health information from different sources. It is also important to shed light on non-users of the internet or people who seldom use information from the internet. How is it possible for them to increase HL when information is mostly digital and therefore these population groups are lacking or not understanding information from public authorities?

## 5. Conclusions

The present study has examined how university college students in Denmark during the COVID-19 pandemic search, find and use digital health-related information by assessing levels of DHL across social characteristics, preferred information-seeking platforms, social media, DHL competences and the impact of help from social networks.

It is evident that an infodemic caused by the COVID-19 pandemic has tremendous consequences for society concerning both individuals and the health system in Denmark. University college students with sufficient DHL are better equipped to avoid misinformation from, e.g., different social media than students with limited HL who often use social media to search for health information.

Our overall results show that most Danish students find it easy to find information on the internet and are rather satisfied with the information they find. However, some students find it difficult to judge the relevance and reliability of the information they find. Students with limited DHL tend to get help more often from their close social network to find and understand information about the COVID-19 pandemic. It is therefore important in future HL research to include the impact of social networks in finding and understanding health information especially for the most vulnerable groups in the society. Research should also develop indicators to measure the impact of social networks on HL and DHL in a quantitative survey design.

Finally, we suggest investment in teacher training and education efforts to increase students’ DHL at universities and university colleges.

## Figures and Tables

**Table 1:** Questions and response options.

	Questions	Response Options
q 15	“When you search the internet for information on the corona virus or related topics, how easy or difficult is it for you to… ”: - make a choice from all the information you find? - use the proper words or search query to find the information you are looking for? - find the exact information you are looking for?	The response options were on a four-point Likert scale (1) very difficult, (2) difficult, (3) easy, and (4) very easy)
q 16	“When you post a message (e.g., on a forum or social media like Facebook or Twitter) about coronavirus or similar topics, how ‘easy’ or ‘difficult’ is it for you to…” - articulate your question or health-related concern clearly? - express your attitude, thoughts, or feelings in writing? - write your message in a way so that people understand exactly what you mean?	The response options were on a four-point Likert scale (1) very difficult, (2) difficult, (3) easy, and (4) very easy)
q 17	“When you search the internet for information on the corona virus or related topics, how easy or difficult is it for you to … ” -check different websites to see whether they provide the same information -decide whether the information is written with commercial interests (e.g., by people trying to sell a product) -decide whether the information is reliable or not	The response options were on a four-point Likert scale (1) very difficult, (2) difficult, (3) easy, and (4) very easy)
q 18	“When you search the internet for information on coronavirus or similar topics, how ‘easy’ or ‘difficult’ is it for you to…” … assess whether the information is useful to you? … use the information you find in your everyday life? … use the information you found to make decisions about your health (e.g., protective measures, hygiene regulations, routes of infection, risks and risk prevention)	The response options were on a four-point Likert scale (1) very difficult, (2) difficult, (3) easy, and (4) very easy)
	Assessment of social network	
q 25	“Do you use your knowledge and skills to help others in your close relationships (e.g., friends, family) to find and understand health information from the internet?”	Yes, I help with finding and understanding relevant information (e.g., about diseases, health issues, and COVID-19) -Yes, I help with advice about, e.g., healthy lifestyle in everyday life (e.g., smoking, nutrition, alcohol, physical activity, and stress/mental well-being) -Yes, I help with assessment of the reliability of health information from authorities (e.g., government, the National Board on Health, region, and municipalities) -No -Not relevant Multiple answers were possible
q 26	“Do you use your knowledge and skills to help others in your close relationships (e.g., friends, family) to find and understand health information from the internet?”	- Yes, often - Yes, sometimes - Yes, but rarely - No - Do not know - Not relevant

**Table 2:** Characteristics of participants (n = 1518).

Characteristics	Total	Female	Male
Participants n (%)	n = 1518	n = 1266	n = 247
Age (years, mean)	28.4 (SD 8.4)	28.3 (SD 8.4)	28.9 (SD 8.1)
Age group			
<26	822 (54.2)	703 (55.5)	115 (46.6)
26–30	287 (18.9)	220 (17.4)	66 (26.7)
>30	409 (26.9)	343 (27.1)	66 (26.7)
Study course	n = 1511 (div n = 5)	n = 1262	n = 244
Education (BA)	751 (49.5)	608 (48.2)	140 (57.4)
Health	470 (31.0)	415 (32.9)	55 (22.5)
Society and administration	264 (17.5)	222 (17.6)	40 (16.4)
Other (vocational diploma? EVU)	26 (1.7)	17 (1.3)	9 (3.7)
Subjective social status *n* (%)	n = 1446 (div n = 5)	n = 1207	n = 234
Low	364 (24.0)	288 (23.9)	72 (30.8)
Medium	952 (62.7)	808 (66.9)	143 (61.1)
High	130 (8.6)	111 (9.2)	19 (8.1)
Level of DHL (excl DHL privacy)	n = 1005	n = 845	n = 157
Sufficient DHL	592 (59.9)	488 (57.8)	101 (64.3)
Limited DHL	413 (41.1)	357 (42.2)	56 (35.7)

**Table 3:** Digital health literacy for each subscale among respondents. N = 1106. Pct.

Characteristics	Very Easy/Easy	Difficult/Very Difficult
Information search		
Make a choice from all the information you find (n = 1106)	84.8	15.2
Use the proper words/search query to find the information you are looking for (n = 1106)	92.7	7.3
Find the exact information you are looking for (n = 1106)	82	18
Adding self-generated content		
Clearly formulate a question (n = 1106)	83.4	16.6
Express opinions, thoughts or feelings in writing (n = 1106)	76	24
Write your message so it can be understood as you intend (n = 1106)	74.4	25.6
Evaluating reliability		
Decide whether the information is reliable or not (n = 1106)	71.9	28.1
Decide whether the information is written with commercial interests (n = 1106)	79.2	20.8
Check different websites to see whether they provide the same information (n = 1106)	83	17
Determining relevance		
Decide whether the information is applicable (n = 1106)	89.1	10.9
Apply the information in daily life (n = 1106)	87.3	12.7
Use information to make health-related decisions (n = 1106)	86.2	13.8

**Table 4:** Help from close relationships (n = 837).

Do You Get Help from Your Close Relationships (e.g., Friends, Family, Classmates) to Find Information about COVID-19?	Overall DHL (Excl Privacy)				
	Limited, n (%)	Sufficient, n (%)	X^2^ (df)	*p*	V
Yes	222 (46.4)	256 (53.6)	9.81 (1)	0.002	0.11
No	128 (35.7)	231 (64.3)			
Yes, often	32 (55.2)	26 (44.8)	11.95 (3)	0.008	0.12
Yes, sometimes	109 (44.7)	135 (55.3)			
Yes, but rarely	81 (46)	95 (54)			
No	128 (35.7)	231 (64.3)			

## Data Availability

The data presented in this study are available upon reasonable request from the corresponding author.
